# Diffusion-weighted imaging for the detection of liver metastases in the preoperative evaluation of pancreatic cancer — are we really at the end of the road?

**DOI:** 10.1007/s00330-023-10159-4

**Published:** 2023-08-21

**Authors:** Timo A. Auer

**Affiliations:** 1 Department of Radiology, Charité – Universitätsmedizin Berlin, corporate member of Freie Universität Berlin, Humboldt-Universität zu Berlin, and Berlin Institute of Health, Charité Campus Virchow (CVK), Augustenburger Platz 1, 13353 Berlin, Germany; 2 Berlin Institute of Health (BIH), Berlin, Germany

The potential for curative treatment in pancreatic cancer depends on two factors: the feasibility of operating on the localized tumor and the absence of distant metastases. During surgery, around 10–20% of patients are deemed unresectable due to unforeseen liver metastasis, peritoneal carcinomatosis, or locally advanced disease [1]. Over 50% of liver metastases occur within the initial 6 months following the surgery [2]. It is presumed that these liver metastases existed at the time of diagnosis but remained undetected before surgery either because they were too small or due to limitations in the imaging technique [1].

CT is the preferred method to evaluate the operability of local findings, thanks to its ability to assess potential vascular infiltration more effectively. On the other hand, MRI surpasses CT in accurately evaluating liver metastases. While the sensitivity of CT ranges from 38 to 76%, the sensitivity of MRI in connection diffusion-weighted imaging (DWI) sensitivity levels between 86 and 97% [1–4]. When discussing liver metastases that go unnoticed or are inaccurately assessed before pancreatic cancer surgery, we typically refer to lesions in the subcentimeter range [1]. In a study that was recently published, colleagues performed a systematic review and meta-analysis to evaluate if MRI with DWI adds additional value compared to contrast-enhanced multiple detector CT (CE-MDCT) alone in the pre-operative evaluation of pancreatic cancer [5]. The study encompassed nine research papers involving a combined 1121 patients, approximately 15% of whom were diagnosed with liver metastases. While achieving sensitivity and specificity rates of 92.4% and 97.3% respectively, the addition of DWI to MRI had the capacity to decrease unnecessary surgical procedures by 6% and led to alterations in medical decisions in nearly 18% of instances [5]. The authors concluded that MRI with DWI may prevent futile surgeries in pancreatic cancer by improving the detection of occult liver metastasis on preoperative CECT with an NNT of 16.6 [5]. Despite DWI having a sensitivity of nearly 90% even for these subcentimeter metastases, a small fraction may still go undetected [1, 3, 4].

In the future, despite the promising performance of DWI, there will still be a certain percentage of futile operations with the associated increase of morbidity, mortality, and costs [1]. The crucial question to address is how to bridge this gap effectively. This might be an opportune moment to explore beyond current horizons and consider other factors where precise detection of liver metastases plays a pivotal role in determining surgical strategies. In the context of resecting colorectal liver metastases (CRLM), disregarding the smallest metastases can lead to dire consequences. After chemotherapy, even the tiniest micrometastases or those that appear to be shrinking (vanishing lesions to be) must be precisely identified and located anatomically. The detection and characterization abilities of MRI can be further augmented by employing hepatobiliary contrast agents, such as gadoxetic acid (gadolinium ethoxybenzyl-diethylenetriamine-penta-acetate; Gd-EOB-DTPA), resulting in an even more widespread use and significant benefits, particularly concerning CRLM. In 2014, Zech et al published the VALUE study, a prospective, randomized trial that compared Gd-EOB-DTPA-MRI, MRI with extracellular contrast media (ECCM-MRI), and CE-MDCT for staging patients with suspected or confirmed CRLM [6]. According to the VALUE study findings, patients who underwent initial diagnostic imaging with Gd-EOB-DTPA-MRI required significantly fewer additional imaging procedures to confidently diagnose and plan treatment compared to those who had ECCM-MRI or CE-MDCT as the initial imaging method [6]. Additionally, Gd-EOB-DTPA-MRI provided a substantially higher level of confidence in the diagnosis and treatment plan compared to the other two modalities. Consequently, Gd-EOB-DTPA-MRI information led to a reduction in the number of patients requiring intra-operative modifications of the surgical plan during liver resection [6]. In 2016, the group around Zech et al even reported a benefit after cost analyses [7]. Other authors, including Koh et al (2012) and Morin et al (2020), have also validated these findings concerning lesion detection in CLRM [8, 9]. It is important to highlight that in real-world scenarios, the increase in accuracy is achieved through a combination of sequences, as demonstrated for example by Koh et al [8].

Now turning our attention back to pancreatic cancer, the question arises whether liver-specific contrast agents can bridge the gap between the high sensitivity of DWI and prevent futile surgeries. The answer is no; liver-specific contrast agents cannot completely close this gap. However, they can certainly narrow it, offering valuable assistance in improving diagnostic accuracy when combining all sequences at hand. Jhaveri et al (2021) compared Gd-EOB-MRI with contrast-enhanced computed tomography (CECT) in the preoperative detection of liver metastasis and its potential to reduce open-close laparotomies in cases of pancreatic ductal adenocarcinoma (PDAC) and concluded that preoperative Gd-EOB-MRI has superior diagnostic performance in detecting liver metastases from PDAC [10]. By providing better information on surgical eligibility, this approach has the potential to decrease the number of futile open-close laparotomies resulting from attempted curative-intent pancreatic cancer surgery [10]. It should be acknowledged that, at this juncture, DWI would have certainly outperformed CT. Amidst mounting economic pressures, employing abbreviated protocols, usually consisting out of three sequences, is one approach to leverage the benefits of more MRI. The combination of a T2-weighted sequence, DWI, and hepatocyte-specific late phase has shown promise due to its compatibility with sensitivity, anatomical allocation, and precise delineation of hepatic cysts [11].

Pancreatic surgery interdisciplinary teams should therefore carefully assess the advantages and disadvantages of adjusting their diagnostic algorithms to minimize unnecessary surgeries caused by undetected or misdiagnosed liver metastases. Cost analyses indicate that apart from the direct benefits to patients, this approach could also result in lower overall costs.

## Declarations

### Guarantor

The scientific guarantor of this publication is PD Dr. Timo Auer

### Statistics and biometry

No complex statistical methods were necessary for this paper.

### Methodology

- Commentary

## References

[1] Litjens G, Riviere DM, van Geenen EJM, Radema SA, Brosens LAA, Prokop M (2020). Diagnostic accuracy of contrast-enhanced diffusion-weighted MRI for liver metastases of pancreatic cancer: towards adequate staging and follow-up of pancreatic cancer - DIA-PANC study: study protocol for an international, multicenter, diagnostic trial. BMC Cancer.

[2] Van den Broeck A, Sergeant G, Ectors N, Van Steenbergen W, Aerts R, Topal B (2009). Patterns of recurrence after curative resection of pancreatic ductal adenocarcinoma. Eur J Surg Oncol.

[3] Holzapfel K, Bruegel M, Eiber M, Ganter C, Schuster T, Heinrich P (2010). Characterization of small (≤10 mm) focal liver lesions: value of respiratory-triggered echo-planar diffusion-weighted MR imaging. Eur J Radiol.

[4] Eiber M, Fingerle AA, Brügel M, Gaa J, Rummeny EJ, Holzapfel K (2012). Detection and classification of focal liver lesions in patients with colorectal cancer: retrospective comparison of diffusion-weighted MR imaging and multi-slice CT. Eur J Radiol.

[5] Altmayer S, Armelin LM, Soares Pereira J et al (2023) MRI with DWI improves detection of liver metastasis and selection of surgical candidates with pancreatic cancer: a systematic review and meta-analysis. Eur Radiol. 10.1007/s00330-023-10069-5 10.1007/s00330-023-10069-5 37566274

[6] Zech CJ, Korpraphong P, Huppertz A, Denecke T, Kim MJ, Tanomkiat W (2014). Randomized multicentre trial of gadoxetic acid-enhanced MRI versus conventional MRI or CT in the staging of colorectal cancer liver metastases. Br J Surg.

[7] Zech CJ, Justo N, Lang A, Ba-Ssalamah A, Kim M-J, Rinde H (2016). Cost evaluation of gadoxetic acid-enhanced magnetic resonance imaging in the diagnosis of colorectal-cancer metastasis in the liver: Results from the VALUE Trial. Eur Radiol.

[8] Koh DM, Collins DJ, Wallace T, Chau I, Riddell AM (2012). Combining diffusion-weighted MRI with Gd-EOB-DTPA-enhanced MRI improves the detection of colorectal liver metastases. Br J Radiol.

[9] Morin C, Drolet S, Daigle C, Deshaies I, Ouellet JF, Ball CG (2020). Additional value of gadoxetic acid-enhanced MRI to conventional extracellular gadolinium-enhanced MRI for the surgical management of colorectal and neuroendocrine liver metastases. HPB (Oxford).

[10] Jhaveri KS, Babaei Jandaghi A, Thipphavong S, Espin-Garcia O, Dodd A (2021). Hutchinson S, et al Can preoperative liver MRI with gadoxetic acid help reduce open-close laparotomies for curative intent pancreatic cancer surgery?. Cancer Imag.

[11] Granata V, Fusco R, Avallone A et al (2020) Abbreviated MRI protocol for colorectal liver metastases: How the radiologist could work in pre surgical setting. PLoS One 15(11):e0241431 10.1371/journal.pone.0241431 PMC7676687 33211702

